# Quality of Life Experienced by Major Lower Extremity Amputees

**DOI:** 10.7759/cureus.17440

**Published:** 2021-08-25

**Authors:** Lemuel Pran, Shanta Baijoo, Dave Harnanan, Hani Slim, Ravi Maharaj, Vijay Naraynsingh

**Affiliations:** 1 Surgery, Eric Williams Medical Sciences Complex, Mt. Hope, TTO; 2 Clinical Surgical Sciences, The University of the West Indies, St. Augustine, TTO; 3 Vascular Surgery, King's College Hospital, London, GBR; 4 Surgery, Medical Associates Hospital, St. Joseph, TTO

**Keywords:** quality of life, major lower limb amputation, prosthesis, trans-tibial amputation, trans-femoral amputation

## Abstract

Lower extremity amputations and diabetic foot-related complications in the Caribbean population have been previously reported. However, there is a lack of evidence that assess the quality of life experienced in such amputees. This study aimed to determine the health-related quality of life (HRQoL) in patients after a major lower limb amputation.

Data collection was performed for all major lower limb amputations undertaken at a tertiary care institution in Trinidad and Tobago, between January 2012 to December 2016. The quality of life for patients who were accessible, alive, and willing to participate was assessed using the EuroQol 5D-5L tool. Statistical analysis was performed using the Mann-Whitney U and Kruskal-Wallis tests comparing medians across various subgroups.

A total of 134 individuals were still alive and willing to participate in the study. The average EQ-5D-5L index value for the cohort was (0.598), which was significantly lower compared to EQ-5D-5L population norms for Trinidad and Tobago p < 0.05. Statistically significant differences were also seen in median EQ-5D-5L index value for patients who ambulated with a prosthesis (0.787) compared to those who used another device for mobilization (0.656), p < 0.05, and to those patients who did not ambulate (0.195), p < 0.05. A comparable Quality of life was seen between the level of amputation (transtibial versus transfemoral) and gender (males versus females), p-values were 0.21 and 1.0, respectively.

Overall quality of life after major amputation, as well as independent mobilization with a prosthesis, continues to be problematic in the Caribbean population. Factors adversely related to the quality of life post major amputation include increasing age, problems related to mobility, and non-ambulatory patients.

## Introduction

Health-related quality of life (HRQoL) in major lower extremity amputees remains an area of concern and thus drives rehabilitation efforts. The primary goals are to ambulate with a prosthesis, enable amputees to perform activities of daily living, and optimize control of co-morbid conditions [1]. Although lower extremity amputations and associated diabetic foot-related complications have been studied in the Caribbean [2-4], there is a paucity of evidence compared to international data which assesses the quality of life experienced by amputees. Health-related quality of life is an area of interest, as many daily functions are impaired, and dependency in these individuals is rampant. Ideally, once the limb stump is adequately healed, an amputee should go on to have a prosthesis within four to eight weeks [1,5]. Additionally, intensive lower limb physiotherapy is required from the early post-operative period extending beyond prosthetic acquisition [1,6-8]. These strategies maintain range of motion at the joint in question, avoid flexion deformities, aid in muscle strengthening; inclusive of the upper limbs (required for self-transfer and ambulation). Mobility and prosthesis acquisition plays a crucial role in the rehabilitation of amputees to pre-morbid functional levels. These two aspects have also been shown to have a significant impact on the quality of life after having a major lower limb amputation [9,10]. Hence, the goal in this population of surgically treated patients should always revolve around the restoration of mobility by employing the various devices available, which are tailored to individual needs. This study aimed to assess the health-related quality of life in patients after major lower limb amputation at a tertiary care institution in Trinidad and Tobago.

## Materials and methods

All major lower limb amputations undertaken at a tertiary care institution in Trinidad and Tobago, between January 2012 to December 2016 were reviewed. A total of 603 amputees were identified, and demographic data were collected. Patients who were younger than 18 years, had a minor lower limb amputation (the level below the ankle) and whose records were not available for review were excluded.

Of these 603 amputees, 134 individuals were found to be still alive and willing to participate in the quality of life (QOL) study. The EuroQol five-level instrument (EQ-5D-5L) was used to assess the quality of life, in addition to demographics, type of major amputation and prosthetic acquisition data was also collected. The assessment was performed as a face-to-face interview in the surgical out-patient clinic by two members of the research team. 

The EQ-5D-5L instrument comprises five dimensions (mobility, activity, self-care, anxiety/depression and pain/discomfort); each dimension has five levels: no problems, slight, moderate, severe and extreme problems. The five-digit numerical code obtained can range from 11111 to 55555 indicating full and worse health respectively [11,12]. The five-digit code obtained was then translated to an index value ranging from 1.000 (best QOL) to 0.000 (worst QOL). The value set for Trinidad and Tobago was developed using the 3 level instrument (EQ-5D-3L). The EQ-5D-5L index values for this study were obtained by applying a crosswalk analysis to the EQ-5D-3L index values [13,14]. The EQ-5D-5L tool also comprises a visual analogue scale (VAS) which is scored 0 (worse health imaginable) to 100 (best health imaginable) by the patient on a subjective basis.

Statistical analysis was performed using the Mann-Whitney U and Kruskal-Wallis tests comparing medians across various subgroups based on age, gender, type of procedure and method of ambulation/ambulation status. Additionally, median values for these subgroups and the cohort were compared to population norm values for EQ-VAS and EQ-5D index. The difference between medians was compared and a p-value less than .05 was considered statistically significant. 

Ethical approval was obtained from the University of the West Indies Ethics Committee (CEC079/11/15) as well as from the relevant Health Authority for conducting this project. Licensed use was obtained from the EuroQol Research Group for the use of their EQ-5D-5L assessment tool for a period of two years commencing November 1st, 2016. 

## Results

Over a five-year period (January 2012 to December 2016) a total of 603 major lower-limb amputations were performed, and demographic data were reviewed. The overall average age was 63 years (range 19 to 100 years) and males accounted for 62% (371/603) of the study group. A transtibial amputation (TTA) was performed in 40% (242/603) and a transfemoral amputation (TFA) 60% (361/603) of the cases Table 1. 

**Table 1:** Demographic data for the overall amputee group and study cohort.

Parameter	Amputee group (n = 603)	Study cohort (n = 134)
Average age (years)	63	63
Average age males (years)	63	62
Average age females (years)	64	63
Transfemoral amputation (%)	60	43
Transtibial amputation (%)	40	57
Males (%)	62	62
Females (%)	38	38

One hundred and thirty-four individuals fulfilled the inclusion criteria for the study and were analysed further. Out of the 134 amputees, 88% (118/134) were diabetic and 60% (80/134) hypertensive. Ischemic heart disease and end-stage renal disease accounted for 15% (20/134) and 7% (9/134) respectively. The mean time at which the quality of life assessment was performed was 42 months (range 6 to 73 months) from the date of amputation. For the majority, the indication for surgery was sepsis in 70% (95/134) and peripheral arterial occlusive disease in 24% (32/134). Amputees who had surgical intervention for trauma and malignancy accounted for 4% and 2%, respectively. 

The EQ 5D-5L index values obtained showed that overall amputees younger than 50- years reported a superior quality of life compared to those older than 50 years (mean rank difference 72/ difference between medians 0.073) p-value: 0.045. Although a higher index value for females younger than 50 years was seen compared to older than 50-year males (mean rank difference 119/difference between medians 0.225), this difference did not reach statistical significance, p-value: 0.059 (Table 2). 

**Table 2:** Quality of life indices based on age and gender.

EQ index	Parameter							
	Males	Females	Overall < 50 years	Overall > 50 years	Males < 50 years	Males > 50 years	Females < 50 years	Females > 50 years
Minimum	0.015	0.002	0.067	0.002	0.067	0.015	0.679	0.002
25% percentile	0.369	0.260	0.730	0.280	0.702	0.321	0.756	0.249
Median	0.743	0.707	0.780	0.707	0.774	0.729	0.798	0.573
75% percentile	0.822	0.827	0.824	0.822	0.822	0.820	0.831	0.826
Maximum	1.000	1.000	0.917	1.000	0.861	1.000	0.917	1.000
Mean rank	271	264	329	257	311	262	367	248

**Figure 1:**
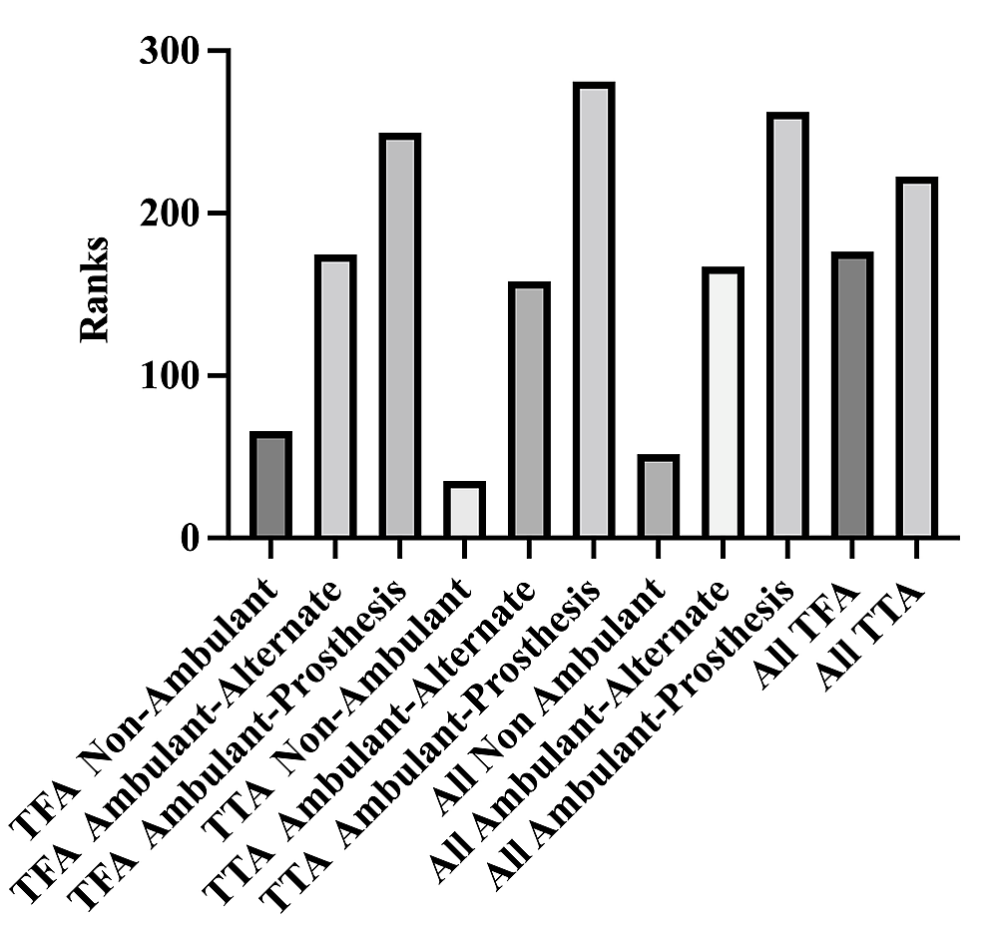
Quality of life indices based on mode of ambulation and type of amputation. TFA: transfemoral amputation; TTA: transtibial amputation.

A higher median index value was obtained for transtibial amputees (TTA) and transfemoral amputees (TFA) combined, who ambulated with a prosthesis than those, who ambulated with an alternate device and who did not ambulate (difference between means 0.131 and 0.592), p-values were < 0.05. Similarly, all amputees (TTA and TFA combined) who ambulated with an alternate form of mobility device versus those who did not ambulate had a higher median index value (difference between medians 0.461), p < 0.05. Transtibial amputees who ambulated with a prosthesis had a higher median index value than those, who did so with an alternate device and those who did not ambulate at all (p-values 0.0001, 0.004 and difference between medians of 0.60 and 0.662, respectively). In the TFA group of patients who did not ambulate a significantly lower index value when compared to those who ambulated with a prosthesis or another device (difference between medians 0.466 and 0.550 respectively), p- values were <.05 shown in Table 3 and Figure 1.

**Table 3:** Quality of life Indices based on mode of ambulation and type of amputation.

Mode of ambulation	Median index value for transtibial amputees (mean rank)	Median index value for transfemoral amputees (mean rank)	Median index value for all amputees (mean rank)
Prosthesis	0.802 (281)	0.777 (215)	0.787 (260)
Alternate device	0.602 (174)	0.693 (195)	0.656 (183)
Non-ambulant	0.140 (35)	0.227 (97)	0.195 (85)
Overall	0.758 (211)	0.702 (186)	0.733 (269)

When the dimensions (mobility, self-care, usual activity, pain/discomfort and anxiety/depression) were individually assessed, mobility as well as usual activity scored worse (mean 3). Regarding other dimensions (self-care, pain/discomfort and anxiety/depression) the frequency of no problems versus any degree of problems were comparable (mean 2), shown in Table 4. 

**Table 4:** Frequency of the five-Level scores for each of the five domains of health assessed.

n = 134	EQ dimensions				
Levels	Mobility	Self-care	Activities	Pain/discomfort	Anxiety/depression
1	31	74	45	63	62
2	37	17	28	42	29
3	15	10	12	18	16
4	5	8	9	9	18
5	46	25	40	2	9
Mean	3	2	3	2	2

**Figure 2:**
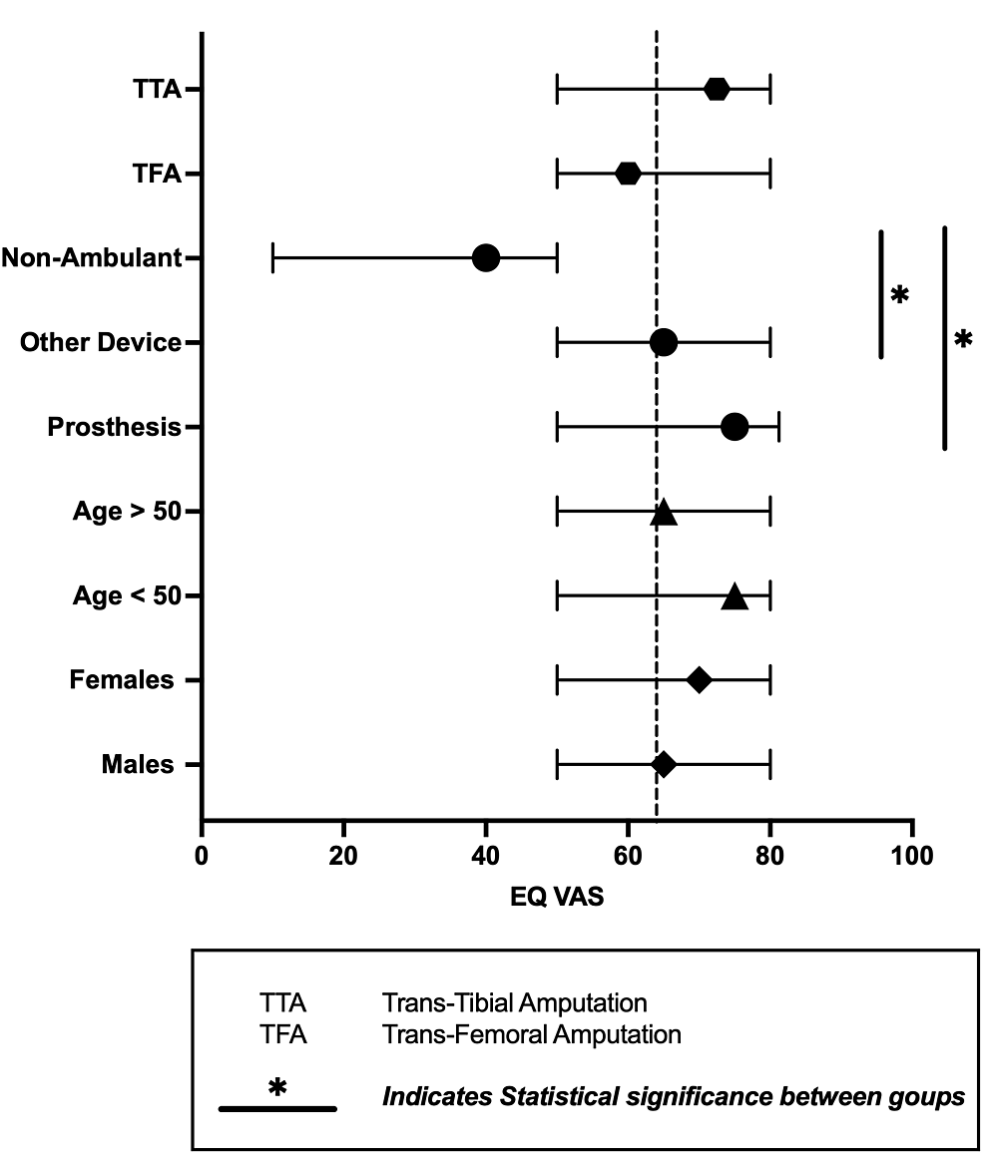
EQ-VAS scores for subgroups based on gender, age, mode of ambulation and type of amputation. EQ-VAS:  EuroQol visual analogue scale.

The EQ-VAS scores ranged from 0 to 100, with a median of 65 (mean rank 336), a mean of 64 with a standard deviation of 21. Sub-group analysis of EQ-VAS showed significantly lower scores for patients who did not ambulate when compared to ambulation with a prosthesis and another device (difference between medians of 35 and 25, respectively) p < 0.05 shown in Figure 2. 

## Discussion

Quality of life for patients who have undergone a major lower limb amputation is an issue of great concern. Hence there has been quite an extensive investigation into this facet of the post-operative experience. The overall quality of life in this study was determined to be below standard when compared to population norms for Trinidad and Tobago [15]. Additionally, individuals who ambulated with a prosthesis were found to have a better quality of life than those who did not. However, no statistically significant difference (a p-value < 0.05) was seen for above versus below-knee amputees. Although this provides some insight into the post-amputation quality of life, caution should be exercised as there can be some misconception. Poor quality of life was observed for the entire cohort, and therefore this is one possible reason no difference was observed between transfemoral and transtibial amputees. Admittedly, many of the countries to which the average overall index values were compared are of the first-world status, with superior health care systems. This cohort had a higher overall index value when compared to Scottish, Swedish and Chinese data [16-18]. Although many of these studies have used the EQ-5D-5L or 3L quality of life assessment tools, there is a variation of the time elapsed post amputation and when the assessment was performed. This poses a significant barrier to comparison as the time to prosthetic acquisition as well as rehabilitation programs varies across all health systems and regions. There exists a considerable degree of disparity regarding demographic, geographic, social and economic factors amongst the populations studied, unfortunately with no real solution. 

Furthermore, there are several quality of life assessment tools that have been developed and used to obtain this information. These include SF-36, WHO BREF, EQ-5D, just to name a few [13,14,19,20]. Due to this high degree of variability amongst the population and the absence of a standard assessment tool, the applicability of findings established internationally cannot be extrapolated to our population [21]. In the Caribbean, there have been several studies that have characterised and placed perspective on major lower limb amputations [2-4]. Several reports highlighting the challenges of limb amputations have emerged from countries such as Barbados, Jamaica and Trinidad [3,22-24]. Despite this, there remains a paucity of data assessing the quality of life experienced by amputees and remains uncharted territory for the Caribbean region. 

The EuroQol 5D-5L assessment tool was used in this study as it was simple, easy to understand, short and assessed the major aspects of quality of life (mobility, self-care, usual activity, pain/discomfort, depression/anxiety and overall health). Several studies have been performed evaluating various populations in an attempt to document standard population utility indices [25-28]. Moreover, this is an international, validated health-related quality of life assessment tool, which includes index values [21]. The real value of using the EQ-5D tool is derived from the existing value indices for Trinidad and Tobago published by Bailey et al. Although the index values were obtained using the EQ-5D-3L, this was easily overcome by translation of the EQ-5D-3L indices into the EQ-5D-5L equivalent [14,15]. There have been several reports of the statistical methods used for this purpose, which have been studied as well as validated internationally and has been deemed acceptable by the EuroQol Research Group [12,13].

This study demonstrated that amputees below the age of 50 years experienced a better quality of life than those over 50 years. These findings are not surprising as Quality of life tends to decrease with advancing age [15]. Furthermore, older patients are more likely to have co-morbid conditions which can all contribute to delayed rehabilitation, affect mobility and therefore result in a poor quality of life. Rehabilitation of the older patient, therefore, needs to be performed in a tailored, aggressive manner, which may require pre-operative conditioning/or selection of these patients. The goals for rehabilitation should, therefore, be set in the pre-operative phase, we strongly believe that all other aspects of care even the surgical intervention necessary should be tailored to this.

Mobility is one of the most critical aspects of quality of life post-amputation, as it is most directly affected, and it influences all other elements. Limb preservation is the single most influential factor on the degree of mobility in a patient having either a minor or major lower limb amputation. The literature is clear and has proposed that having a TTA is more favourable as mobility, attaining a prosthesis is more likely, and less energy is required when compared to a TFA [29,30]. Although the median index value (0.758) for patients with a TTA was higher than the mean index value (0.702) for patients with a TFA this difference was not significant (p-value = 0.21). This finding can be explained by the fact that the amputee population in the study had a poor quality of life overall and therefore, differences between the two groups were indistinguishable. 

Further subgroup analysis revealed that the method of ambulation impacted significantly the quality of life. Quality of life index values for amputees who ambulated with a prosthesis (overall, TTA and TFA, 0.787) were higher than alternative modes of ambulation (0.656) as well as non-ambulant patients (0.195), p-value: 0.0004 and 0.0001, respectively. Additionally, those amputees who were non-ambulant had significantly worse quality of life than the other two groups who ambulated. Based on this information, we can therefore infer that the ideal mode of ambulation for an amputee is with a prosthesis. Furthermore, mobilisation with another device such as a Zimmer frame, crutches or a wheelchair, although not ideal is superior to not ambulating at all. There is strong evidence in the literature to suggest that ambulating with a prosthesis is the most influential factor on an amputee's quality of life [10]. Hence adaptation of a similar approach to major lower limb amputation as proposed by the Vascular Society of the United Kingdom in their guidelines for holistic care is a step in the right direction [8]. One of the primary goals of this programme which involves a multi-disciplinary team, with multiple stages of interventions (pre-operative to rehabilitation), was to reduce the 90-day mortality rate to less than 10%. There have also been other reports which have emphasized a multi-staged, multi-disciplinary approach in an attempt to achieve better outcomes of quality of life [1].

To date, this study is one of few evaluating the quality of life in major lower limb amputees emerging from the English-speaking Caribbean. Several factors have been identified such as younger age, ability to ambulation and prosthetic use is associated with a better quality of life. Hence this data can be used to change the approach to major lower limb amputees locally and regionally. Furthermore, the use of the EuroQol 5D-5L tool is of value in the amputee population. The authors would recommend including it prospectively in the future for any amputee to address issues that may arise in the five domains of health-related quality of life. This will allow these issues to be addressed promptly and achieve better outcomes. Additionally, this study can pave the way for further investigation into health-related quality of life in other sub-groups of surgically treated patients.

## Conclusions

Major limb amputations continue to affect the Caribbean population significantly. The overall quality of life after major amputation, as well as independent mobilization with a prosthesis, continues to be problematic in this population. Factors adversely related to the quality of life post major amputation include increasing age and non-ambulatory patients. Ambulating with a prosthesis in patients with either a transtibial and transfemoral was found to be associated with a better quality of life.
